# Supplementary material for: Potential Association of Cytochrome P450 Copy Number Alteration in Tumour with Chemotherapy Resistance in Lung Adenocarcinoma Patients
Source: Int J Mol Sci. 2023 Aug 29;24(17):13380. doi: 10.3390/ijms241713380 (PMC10487787; doi:10.3390/ijms241713380)
Supplement: Supplementary file 1 [file ijms-24-13380-s001.zip › ijms-2564706-supplementary.pdf]

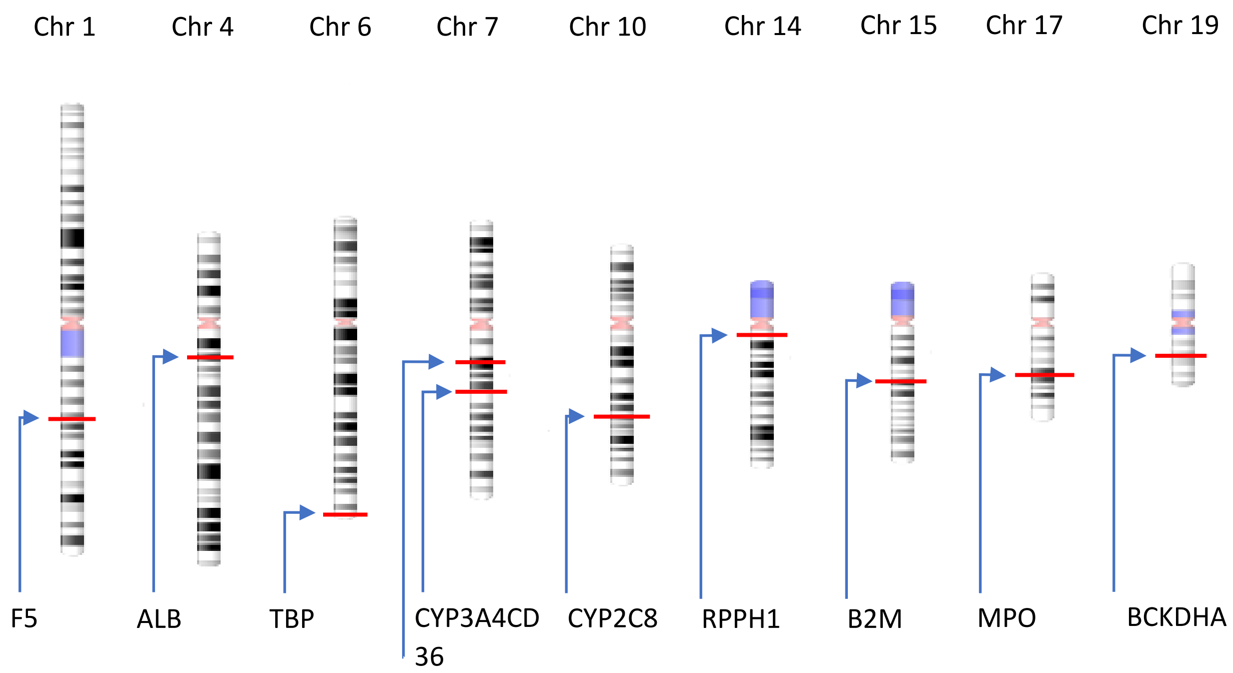

Supplementary Figure S1: Chromosomal localization of the genes evaluated in the present study using the National Center for Biotechnology Information (NCBI) Genome Decoration Page human GRCh38| 850 cytogenes. Chromosomes were aligned to their centromeres. Pink indicates the centromere of the chromosome, while the red line and blue arrow indicate the locations of the selected genes within the chromosome arm.

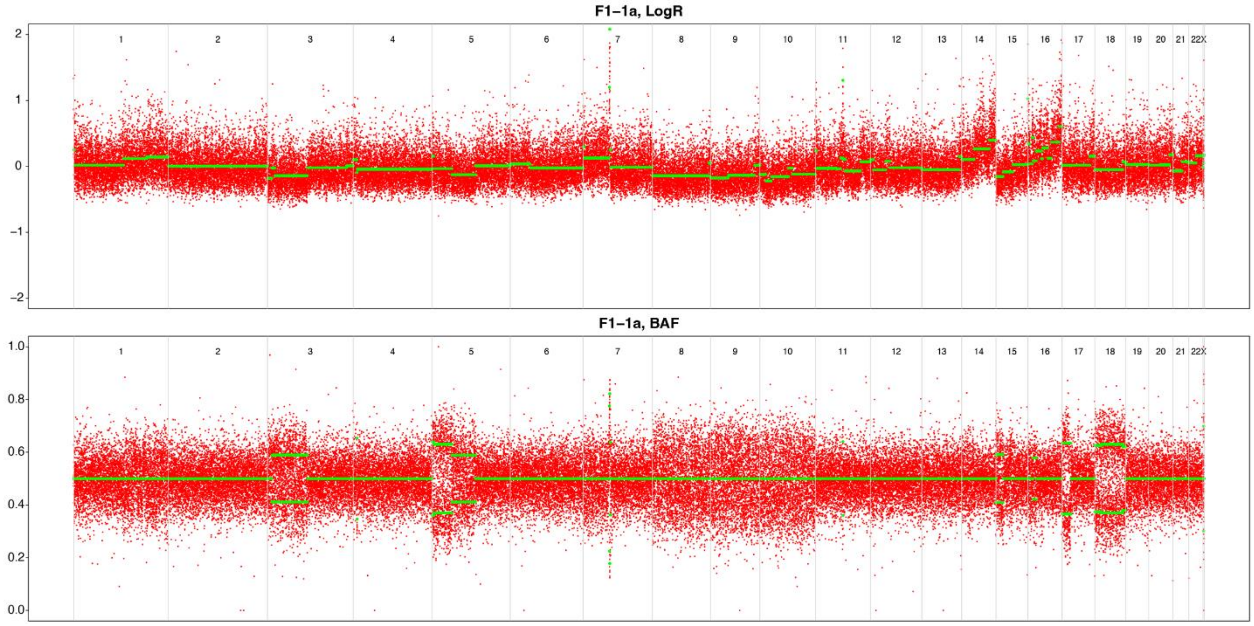

Supplementary Figure S2: Segmented coverage and B allele frequency profiles of the primary lung cancer sample used for validation. The individual red points show log normalized coverage values (top panel) and allele frequencies (bottom panel), determined from a whole genome sequencing datasets, using a list of common human SNPs. The green lines are piecewise constant fitting segmentations, obtained by running the ASCAT pipeline.

Supplementary Table S1: Relative copy numbers of the genes in primary lung adenocarcinoma, lung and liver metastasis.

Gene	Primary lung adenocarcinoma	Lung metastasis	Liver metastasis
RPPH1	1.421	0.976	1.791
ALB	0.904	0.662	0.601
B2M	0.925	1.029	0.812
BCKDHA	1.135	1.426	1.348
CD36	1.038	1.132	1.289
F5	1.313	1.402	2.166
MPO	0.860	1.035	0.916
TBP	1.049	0.935	1.096
CYP3A4	1.137	0.959	0.890
CYP2C8	0.843	0.672	0.577

Supplementary Table S2: Oligonucleotide sequences for *CYP2C8*, *CYP3A4* and *CYP3A5* SNP analysis

Allele (SNP)	Oligonucleotide	Sequences (5’ -> 3 ‘)
CYP2C8*3 **(2130G>A)**	Forward	CGT TTC TCC CTC ACA ACC TTG C
	Reverse	ACT GTT AAG GTC AAT GAC GCA GA
	Probe - wild	CCT CAA TGC TC**C** TCT TCC CCA TCC CA
	Probe - variant	TCC TCA ATG CTC **T**TC TTC CCC ATC CCA
CYP2C8*3 **(30411A>G)**	Forward	CGC TAC GTG ATG TCC ACT AC
	Reverse	GCT GAG AAA GGC ATG AAG TAG T
	Probe - wild	CGT GCT ACA TGA TGA CA**A** AGA ATT TCC TA
	Probe - variant	CGT GCT ACA TGA TGA CA**G** AGA ATT TCC TA
CYP2C8*4 **(11041C>G)**	Forward	TCC CAG GAA CTC ACA ACA AAG T
	Reverse	CGA TGA ATC ACA AAA TGG ACA AGA
	Probe - wild	CGG GAC TTT AT**C** GAT TGC TTC CTG ATC
	Probe - variant	CGG GAC TTT AT**G** GAT TGC TTC CTG ATC
CYP3A4*1B **(-392A>G)**	Forward	TCT GTA GGT GTG GCT TGT TG
	Reverse	AAG GGT TCT GGG TTC TTA TCA G
	Probe - wild	TCG CCT CTC TC**T** TGC CCT TGT
	Probe - variant	TCG CCT CTC TC**C** TGC CCT TG
CYP3A4*22 **(15389C>T)**	Forward	CAG AGG TAG GTC TAA TTC AGT TCA
	Reverse	AGA TCA CCT TCT ATC ACA CTC C
	Probe - wild	ATC ACA CCC AG**C** GTA GGG C
	Probe - variant	ATC ACA CCC AG**T** GTA GGG CC
CYP3A5*3 **(6986A>G)**	Forward	GAG AGT GGC ATA GGA GAT ACC
	Reverse	TGT ACG ACA CAC AGC AAC C
	Probe - wild	TTT GTC TTT CA**A** TAT CTC TTC CCT GT
	Probe - variant	TTT GTC TTT CA**G** TAT CTC TTC CCT GT
